# Supplementary material for: Comparative Transcriptome and Metabolome Profiling Reveal Mechanisms of Red Leaf Color Fading in Populus × euramericana cv. ‘Zhonghuahongye’
Source: Plants (Basel). 2023 Oct 9;12(19):3511. doi: 10.3390/plants12193511 (PMC10575148; doi:10.3390/plants12193511)
Supplement: Supplementary file 1 [file plants-12-03511-s001.zip › plants-2606248-supplementary.pdf]

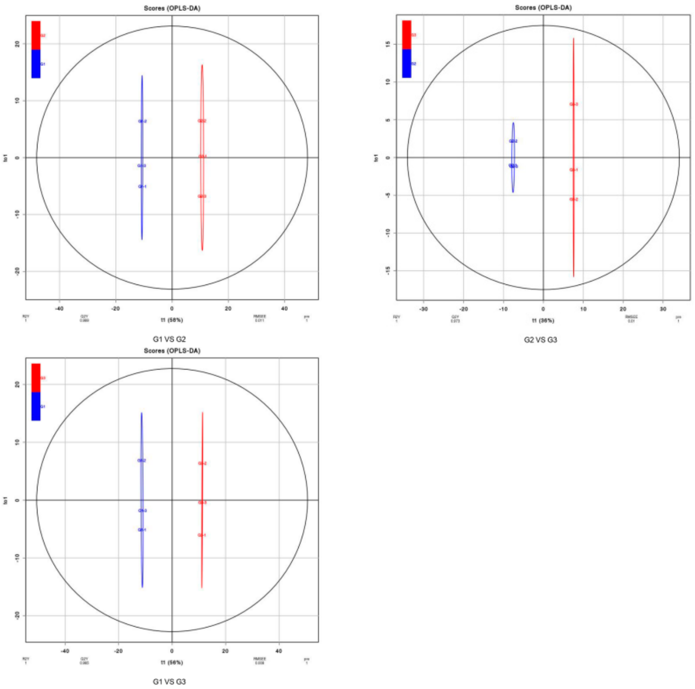

Figure S1. Leaf metabolite OPLS-DA score plot at different developmental stages.

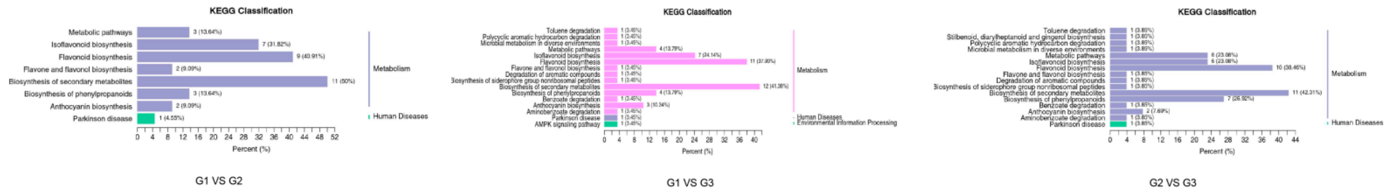

(a)

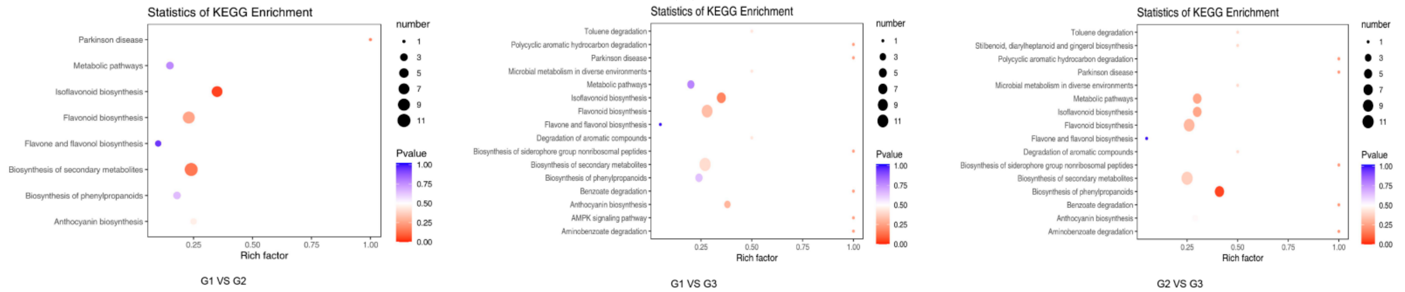

(b)

Figure S2. KEGG map of differential metabolites: (**a**) Differential metabolite KEGG classification chart; (**b**) Differential metabolite KEGG enrichment plot.

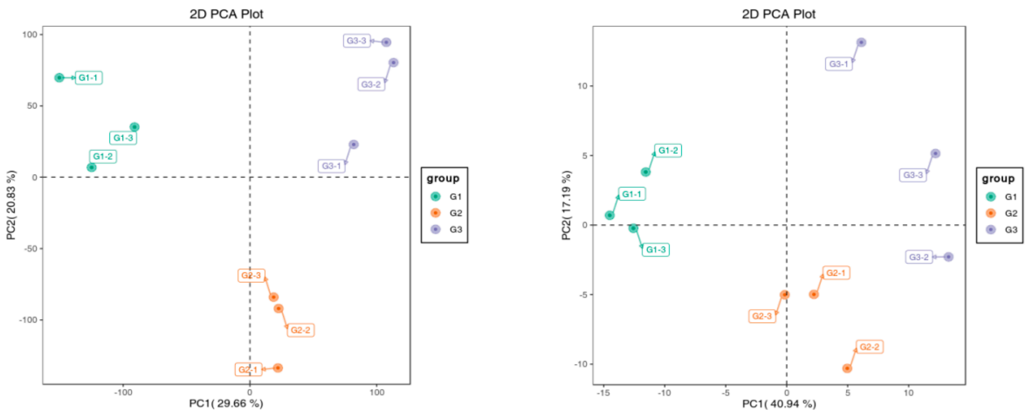

(**a**) (**b**)

Figure S3. PCA analysis: (**a**) Metabolome PCA analysis diagram ; (**b**) Transcriptome PCA analysis diagram. The horizontal coordinate table represents principal component 1, the vertical coordinate represents principal component 2, and the dots of different colors represent samples of different groups.

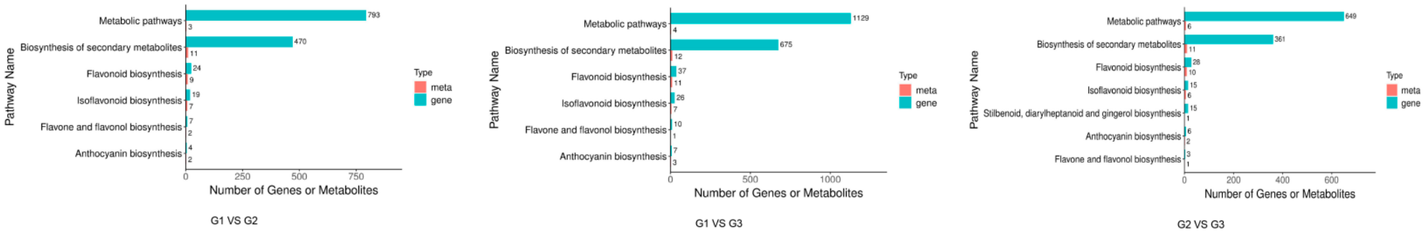

(**a**)

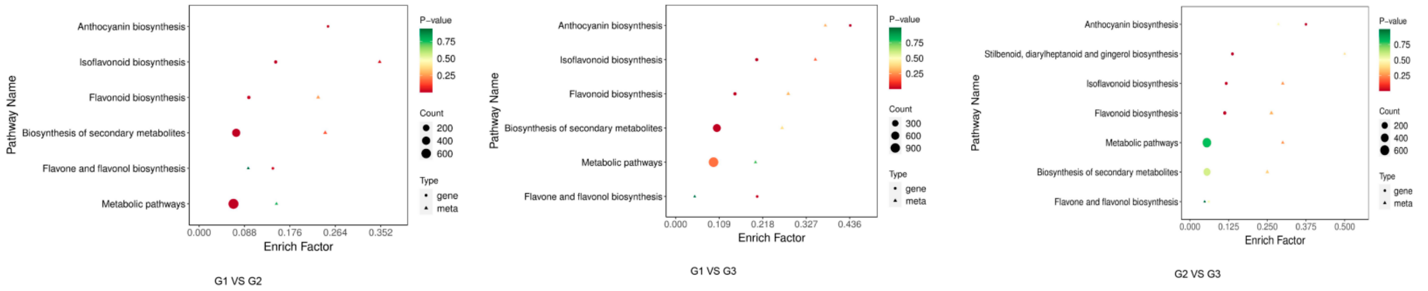

(**b**)

Figure S4. Association analysis based on KEGG: (**a**) KEGG enrichment analysis bar chart; (**b**) KEGG enrichment bubble diagram.

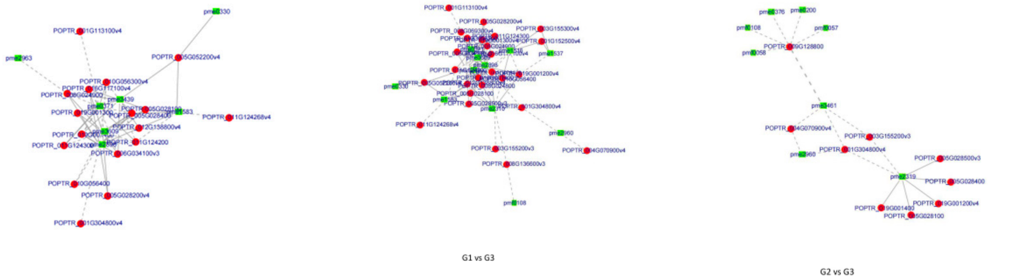

Figure S5. Correlation network diagram.

Note: Metabolites are marked in green squares and genes in red circles. The solid line represents the positive correlation and the dashed line represents the negative correlation.

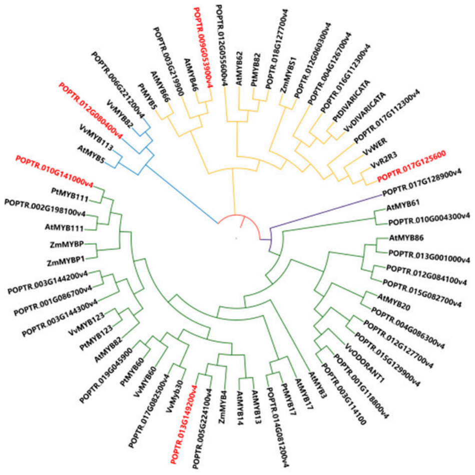

Figure S6. Phylogenetic tree of the *MYB* transcription factor that related to regulation of flavonoids and anthocyanin biosynthesis in'Zhonghuahongye'.

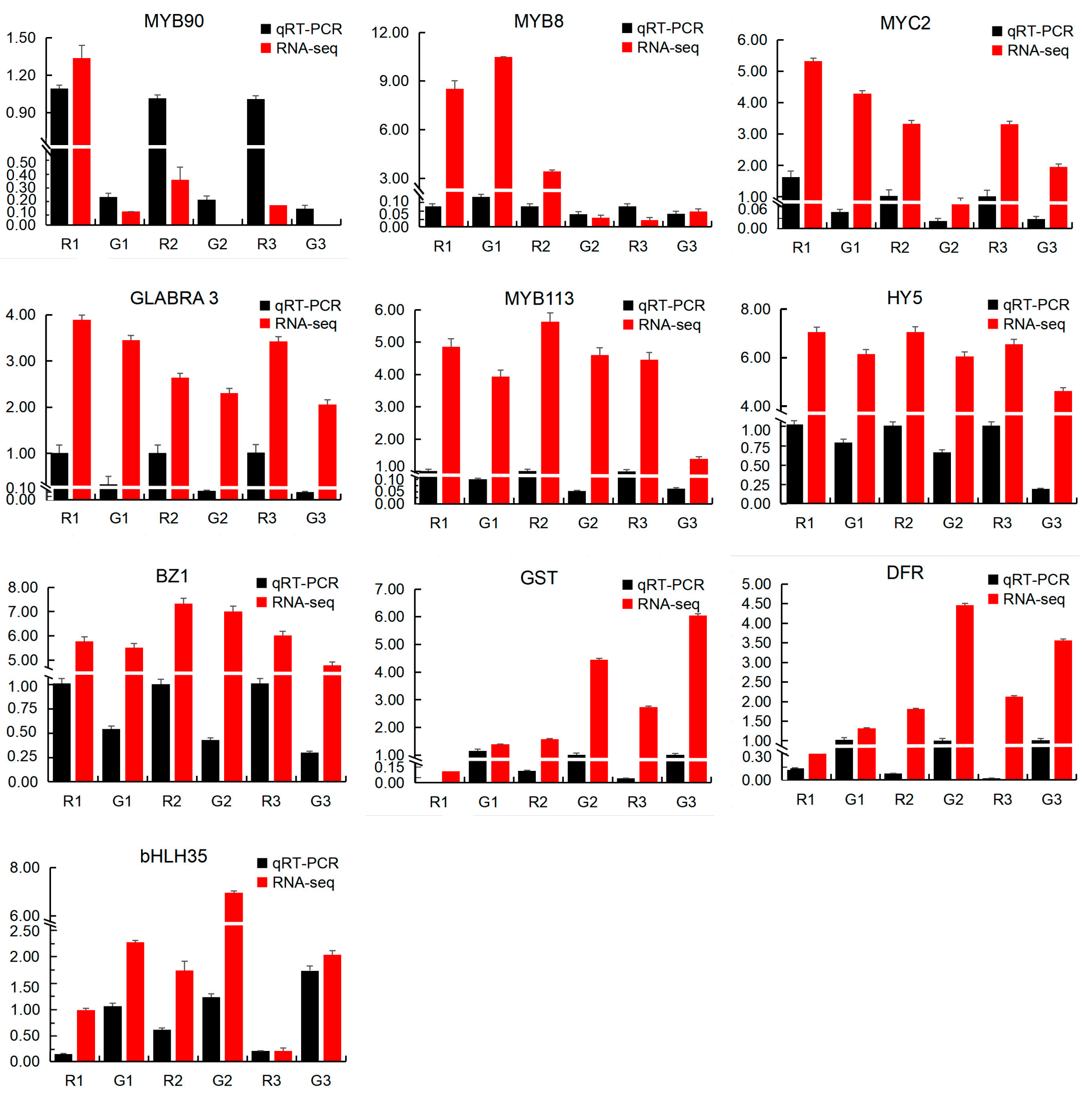

Figure S7. Real-time florescence quantitative results.

Table S1. Primers used in qRT-PCR.

Gene Name	Gene ID	Forward primer	Reverse primer

bHLH35	POPTR_018G141700v4	TAAGCCAACCATCTCTTCC	ACTTATATAACAAACTATATGGGCTG
HY5	POPTR_018G029500v4	TAATGAGAGAAACATGATGCAC	AAATTTCAATAAAGGGGTCCA
MYB90	POPTR_017G125800v4	CTGCTATCTGGTTTGCCTC	TTAATTGTGAACATGCTGCCTA
MYB8	POPTR_018G049600v4	CCGTGTGATTTCTTCAACA	ACTCTTTCTGATTGTGCAT
MYC2	POPTR_002G176900v4	TGAGTAAAACCAGCAAATAGCAG	TAAAAGCTAATGGAACAGGCAAC
GLABRA 3	POPTR_001G103600v4	CACTACAGAGAAATGGACCCGAT	TTTCTAGTCCATAGCTTGCCCAGA
MYB113	POPTR_017G125600v4	TGACTATGCTGCTACTACCG	TAATTTAATAAGTTGCGCTATGGG
DFR（putative anthocyanidin reductase）	POPTR_003G138400v4	AGATTGCGTTAGGAATTCTACCAC	GAGTCTTCAACAGCCAATACCAG
BZ1（anthocyanidin 3-*O*-glucosyltransferase 7）	POPTR_013G143900v4	ATAGATATAGCCTTCCTACCAG	GCCACAGAATTAGATTTCTCCC
GST（probable glutathione S-transferase parC）	POPTR_011G113125v4	TTAGAGTTTTAGGTTTCGGGTAT	AACCCGAAACCCTAAACCAC

Table S2. Significant correlation between differential metabolites and differential genes regulating anthocyanin synthesis of 'Zhonghuahongye'.

Metabolites	Gene ID	Correlation
Naringenin 7-*O*-glucoside (Prunin)(pme0371)	POPTR_005G028400	-
	POPTR_008G024900	-
	POPTR_001G113100v4	-
	POPTR_010G056300v4	+
Dihydromyricetin(pme2898)	POPTR_010G056400	-
	POPTR_001G304800v4	-
Hesperetin(pme2319)	POPTR_005G028400	+
	POPTR_005G028100	+
	POPTR_019G001400	+
	POPTR_019G001200v4	+

Note: -: negative correlated; +: positive correlated.

Table S3. Enzymes bioanabolically associated with anthocyanins of 'Zhonghuahongye'.

Full name	Abbreviation
UDP-glucose:flavonoid 3-*O*-glucosyltransferase	3GT
4-Coumarate:CoA ligase	4CL
Anthocyanin synthase	ANS
Cinnamate 4-Hydroxylase	C4H
Chalcone isomerase	CHI
Chalcone reductase	CHR
Chalcone synthase	CHS
Dihydroflavonol 4-reductase	DFR

Flavonoid-3′,5′-hydroxylase	F3′5′H
Flavonoid-3′-hydroxylase	F3′H
Flavanone 3-hydroxylase	F3H
Flavonol synthase	FLS
Ferredoxin-NADP+ oxidoreductase	FNR
Glycosyl transferases	GT
Isoflavone synthase	IFS
Leucoanthocyanidin dioxygenase	LDOX
O -methyltransferase	OMT
Phenylalaninammo－Nialyase	PAL
UDP-glycose flavonoid glycosyltransferase	UFGT

Table S4. Differentially expressed genes.

POPTR_006G126800v4	POPTR_010G223900v4	POPTR_010G224200
POPTR_001G036900v4	POPTR_003G188500	POPTR_004G082000v4
POPTR_004G102000v4	POPTR_006G036200v4	POPTR_006G169700
POPTR_001G051500	POPTR_002G141400v3	POPTR_009G128800
POPTR_012G138800v4	POPTR_005G028100	POPTR_005G028400
POPTR_005G028500v3	POPTR_019G001200v4	POPTR_019G001400
POPTR_001G371900	POPTR_002G125400v4	POPTR_009G133300v4
POPTR_001G113100v4	POPTR_001G165200	POPTR_019G078400v4
POPTR_019G087300v4	POPTR_019G108900v4	POPTR_011G047800
POPTR_001G113100v4	POPTR_003G119100v4	POPTR_006G101100v4
POPTR_016G117100v4	POPTR_001G304800v4	POPTR_004G050500v4
POPTR_008G136600v3	POPTR_011G059500v4	POPTR_012G006400v4
POPTR_013G121300v4	POPTR_013G122900	POPTR_013G129500v4
POPTR_019G093000v4	POPTR_019G093200v3	POPTR_001G113100v4
POPTR_003G119100v4	POPTR_006G101100v4	POPTR_008G069300v4
POPTR_016G117100v4	POPTR_003G155200v3	POPTR_003G155300v4
POPTR_006G219600v4	POPTR_001G152500v4	POPTR_003G155300v4
POPTR_005G028200v4	POPTR_006G034100v3	POPTR_008G069300v4
POPTR_011G124300	POPTR_001G023800	POPTR_001G214600v4
POPTR_006G195300v4	POPTR_016G060700	POPTR_010G056300v4
POPTR_010G056400	POPTR_011G124268v4	POPTR_013G027000v4
POPTR_001G113100v4	POPTR_005G028400	POPTR_008G024900
POPTR_009G128800	POPTR_010G056300v4	POPTR_009G069100
POPTR_001G333400	POPTR_001G304800v4	POPTR_010G056400
POPTR_004G139700v4	POPTR_002G125400v4	POPTR_010G125400v3
POPTR_001G113100v4	POPTR_003G119100v4	POPTR_006G069600v4
POPTR_019G061700v4	POPTR_019G071200v3	POPTR_008G116500v4
POPTR_013G121400	POPTR_013G122100v4	
